# Effects of topical hypothermia on postoperative inflammatory markers in patients undergoing coronary artery bypass surgery

**DOI:** 10.5830/CVJA-2014-005

**Published:** 2014-04

**Authors:** Murat Kadan, Gokhan Erol, Bilgehan Savas Oz, Mehmet Arslan

**Affiliations:** Department of Cardiovascular Surgery, Gulhane Military Academy of Medicine, Etlik, Ankara, Turkey; Department of Cardiovascular Surgery, Gulhane Military Academy of Medicine, Etlik, Ankara, Turkey; Department of Cardiovascular Surgery, Gulhane Military Academy of Medicine, Etlik, Ankara, Turkey; Department of Cardiovascular Surgery, Gulhane Military Academy of Medicine, Etlik, Ankara, Turkey

**Keywords:** cardiopulmonary bypass, hypothermia, topical cooling, diaphragmatic paralysis, postoperative atrial fibrillation

## Abstract

**Background:**

We aimed to examine the effects of topical hypothermia on inflammatory markers in patients undergoing coronary artery bypass surgery.

**Methods:**

Fifty patients undergoing isolated coronary artery bypass surgery were included the study. They were randomised to two groups. Mild hypothermic cardiopulmonary bypass (28–32°C) was performed on both groups using standardised anaesthesiology and surgical techniques. Furthermore, topical cooling with 4°C saline was performed on patients in group I. We recorded peri-operative and intra-operative results of blood samples, pre-operative and postoperative outcomes of electrocardiography and echocardiography, diaphragm levels on X-ray, and the necessity of positive inotropic medication and intra-aortic balloon pump (IABP).

**Results:**

Time-dependent changes in blood samples were compared between the two groups. The changes on complement 3 (C3) and TNF-α levels were more significant in group I than group II (*p* < 0.05 and *p* < 0.001, respectively). Spontaneous restoration rate of sinus rhythm was higher in group II than group I (80 vs 32%, *p* < 0.01). Atrial fibrillation was seen in six patients in group I and one patient in group II (*p* < 0.05). IABP was performed on four patients (16%) in group I (*p* < 0.05). Diaphragmatic paralysis was seen in seven patients in group I but not in group II (*p* < 0.01). Partial pericardiotomy rates were compared within the groups but there was no statistically significant difference (*p* > 0.05). One patient in group I died on the 18th postoperative day, but operative mortality rate was not statistically significant between the two groups (*p* > 0.05).

**Conclusions:**

Topical hypothermia had a negative impact on inflammatory markers and postoperative morbidities.

## Abstract

Since Frey and Gruber’s theory in 1885 of circulating blood through a machine in order to pump and oxygenate it, cardiopulmonary bypass (CPB) has improved dramatically and has become almost indispensable in cardiac surgery today.^1^ However, there are many consequences such as inflammation and thrombosis.^2^ The notion that by changing the body temperature we could decrease inflammatory activity and thus mortality and morbidity rates has intrigued cardiac surgeons over time.

Topical and systemic hypothermia is applied in many centres today but there is little data on the advantages and disadvantages of topical cooling. We aimed to investigate the effects of topical hypothermia on postoperative cardiac function in patients undergoing coronary artery bypass surgery.

## Methods

This study was approved by the ethics committee of Gulhane Military Academy of Medicine. Informed consent was obtained from all patients involved.

Fifty-four patients diagnosed with coronary artery disease using coronary angiography (CAG) and who were not suitable for percutaneous or minimally invasive treatment techniques were included. The exclusion criteria included previous heart or pulmonary surgery, emergency revascularisation (within the first 24 hours after CAG), concomitant cardiac surgical procedures (valve repair or replacement, atrial or ventricular septal repair, aneurysmectomy, coronary endarterectomy etc.), left ventricular aneurysm diagnosed by echocardiography or angiography, myocardial infarction within the last two weeks, low ejection fraction (EF) (< 35%), and the necessity for pre-operative intra-aortic balloon pump (IABP), temporary or permanent pacemaker, and positive inotropic pharmacological drugs. Four patients who had ungraftable coronary arteries intra-operatively were also excluded from the study in order to obtain optimum standardisation.

The remaining 50 patients were randomised into two groups. Group I patients included those undergoing surgery using 4°C saline for topical hypothermia and mild hypothermic CPB (28–32°C) (*n* = 25). Group II patients were to undergo surgery without topical hypothermia but mild hypothermic CPB (28–32°C) (*n* = 25).

A median sternotomy was performed on all patients after general anaesthesia. Standard right atrial cannulation with two-staged venous cannula and aortic cannulation was performed after left internal mammarian artery (LIMA) harvesting (if it was to be used). A roller pump (Ann Arbor, Michigan, USA) and membrane oxygenator (Dideco Evo adult fiber oxygenator, Dideco, Mirandola, Italy) were used with mild hypothermia (28–32°C). The patient’s body temperature was measured with a rectal probe. Anticoagulation was provided at a dosage of 300 U/kg unfractionated heparin sulphate.

Blood samples were collected for baseline evaluation in both groups from the coronary sinus via a retrograde cardioplegia cannula just before aortic cross clamping (ACC). Blood cardioplegia solutions at 20°C were delivered for initial cardioplegia in both antegrade and retrograde manner. Topical hypothermia was maintained with 1 000 ml of 4°C saline with induction of cardioplegia in patients in group I only. Maintenance cardioplegia (22°C blood cardioplegia solution) was delivered from the antegrade cannula at 20-minute intervals without topical cooling in both groups.

Blood samples were collected again from the coronary sinus for evaluation of ischaemia just before maintenance cardioplegia was delivered in both groups. Blood cardioplegia solutions at 32°C were delivered after completion of the distal anastomosis, and then the ACC was removed. Topical myocardial rewarming was provided with 36°C saline in group I patients. Spontaneous defibrillation, dysrhythmias, and the necessity for defibrillation and pacemaker implantation were recorded at this time.

The last blood samples for evaluation of reperfusion were collected inside the retrograde cannula in both groups, and then CPB was stopped after the body temperature reached 36°C. After neutralisation of anticoagulation, standard procedures such as control of bleeding, placing of pacemaker wires, and insertion of drainage tubes were performed. The operation was terminated with standard surgical techniques and the patients were transported to the intensive care unit (ICU).

Maximum care was taken to avoid phrenic nerve injury during LIMA harvesting. The first intercostal artery was devascularised with haemostatic clips without cauterisation. Partial pericardiotomy was avoided as far as possible to prevent phrenic nerve injury. Patients who underwent cauterisation or partial pericardiotomy despite these protective methods were also recorded. Standard surgical procedures prevailed throughout the study

Routine pre-operative examinations were done. Echocardiographic evaluation was performed on all patients pre-operatively and just before discharge by the same cardiologist who was blinded to the patient population. Electrocardiograms were taken with the same device pre-operatively, at the 24th hour postoperatively and just before discharge.

Troponin I (TnI), troponin T (TnT), myoglobin, CK-MB and lactate dehydrogenase (LDH) levels were assayed from blood samples, which were collected from the peripheral venous system pre-operatively and at the eighth and 24th hours of ACC. CK-MB and LDH levels were measured with spectrophotometric methods using an Olympus AU640 (Shizuoka-ken, Japan) device, myoglobin and TnI levels were measured using chemiluminescence on a Backman Coulter Access II (Fullerton CA, USA) device, and TnT levels were measured with electrochemiluminescence methods on a Roche Elecys 2010 (Tokyo, Japan) device.

Thoracic X-rays were taken from the same machine (AMX-4 plus, General Electric Company, NWL Bordentown NJ, USA), at the same distance, with the same dosage for individual patients and with the same technician pre-operatively, 48 hours postoperatively and before discharge. Diaphragm levels, pleural effusions and other possible complications were recorded.

TnI, TnT, myoglobin, complement 3 (C3), C4 and TNF-α levels were examined from blood samples, which were taken from the coronary sinus via the retrograde cardioplegia cannula for basal, ischaemia and reperfusion analysis as mentioned above. We aimed to determine the cardiac myocyte reserves directly by analysing myocardial enzymes and complement factors at different periods of ischaemia. C3 and C4 levels were measured with nephelometric methods, using Dade Behring BN II kits (Siemens, Germany), and TNF-α levels were measured with Elisa methods, using the Human TNF-alpha instant ELISA kits (e Bioscience, USA).

Other parameters such as ACC time, total CPB time, cardioplegia amounts, number of proximal and distal anastomoses, necessity for defibrillation and pacemaker placement, and necessity for IABP and/or positive inotropic agents were recorded. Endotracheal intubation time, ICU length of stay, total drainage and transfusion amounts, dysrhythmias, necessity for re-operation, length of hospital stay, and existence of pleural effusion or diaphragmatic paralysis were also recorded.

The patients were followed up on the first week and first month of discharge. ECGs were evaluated by the same cardiologist who was unaware of the patient population. New development of ischaemia-specific changes, such as ST-segment elevations, Q waves, Pardee waves and bundle branch blocks on ECG were seen as abnormal changes in myocardial function, while negative T waves were seen as pericardial reactions.

The X-rays of patients were evaluated by a radiologist who was blinded to the patient population. Necessity for drainage and amounts of pleural effusions, diaphragm paralysis and elevations were recorded. Diaphragm changes above two or more ribs were determined as a positive result, according to the current literature data.^3^ Positive echocardiographic changes were determined as follows: new development of valve disorders, structural or transactional changes of the ventricular wall, aneurysm formation and a decrease in EF of more than 10%.

In addition, haemodynamic parameters, respiratory parameters, blood gas levels, drainage amounts, muscle strength and body temperature were recorded for decisions on extubation time. Intubation time was recorded from the first intubation point in the operating room to the extubation point in ICU. All patients were discharged from ICU to clinical service after removal of their drainage tubes. Operative mortality was evaluated as mortality within the first 30 days.

## Statistical analysis

This was performed with the package program SPSS for Windows 15.0. Chi-square and Fisher’s exact tests were used for comparisons of qualitative data for both groups, and the *t*-test was used for comparisons of quantitative data of free samples. The paired *t*-test was used for quantitative data analysis of time-dependent changes. Assessment of time-dependent changes of inter-group differences was done with two-way ANOVA for repeated measurements.

Frequency and percentage data were used as a descriptive value for qualitative data, and arithmetic mean ± standard deviation as quantitative data. A *p*-value < 0.05 was considered statistically significant.

## Results

Fifty patients (42 male and eight female) were included in this study. The mean age of the patients was 62.8 ± 9.9 years (40–80) in group I, and 57.7 ± 9.4 years (39–84) in group II. With regard to demographic data of the patients, there was no significant difference between the groups other than hypertension. The pre-operative demographic data are given in Table 1.

**Table 1:** Pre-operative demographic data of the patients.

*Demographics*	*Group I (n = 25)*	*Group II (n = 25)*	p*-value*
Age (year)	62.8 ± 9.9	57.7 ± 9.4	> 0.05
Gender (male/female)	22/3	20/5	> 0.05
Height (cm)	169.8 ± 6.52	166.24 ± 6.11	> 0.05
Weight (kg)	76.4 ± 9.03	78.4 ± 9.93	> 0.05
BSA	1.868 ± 0.11	1.852 ± 0.11	> 0.05
DM (*n*)	6	8	> 0.05
HT (*n*)	9	16	< 0.05
Dyslipidaemia (*n*)	8	10	> 0.05
PAD (*n*)	1	1	> 0.05
COPD (*n*)	2	4	> 0.05
Renal failure (*n*)	0	1	> 0.05
Pre-operative EF (%)	55.4 ± 7.79	54.6 ± 7.02	> 0.05

There was no statistically significant difference between the groups with regard to surgical data (*p* > 0.05). Mean aortic cross-clamp time was 55.76 ± 23.2 min in group I, and 53.44 ± 16.7 min in group II. The LIMA was used in 19 patients in group I and in 20 patients in group II. The right coronary artery was revascularised in 14 patients in both groups. Surgical data are given in Table 2.

**Table 2:** Intra-operative data of the patients.

	*Group I (n = 25)*	*Group II (n = 25)*	*p-value*
X clamp time (min)	55.76 ± 23.2	53.44 ± 16.7	> 0.05
Total perfusion time (min)	106.44 ± 40.5	104.28 ± 28.06	> 0.05
Hypothermia (°C)	28.7 ± 0.93	28.8 ± 0.92	> 0.05
Cold cardioplegia (ml)	1035.6 ± 360.17	1014 ± 242.2	> 0.05
Hot cardioplegia (ml)	398 ± 82.25	414 ± 70	> 0.05
Distal anastomosis (*n*)	3 (2–6)	3 (2–5)	> 0.05
Proximal anastomosis (*n*)	2 (1–4)	2 (1–5)	> 0.05
LIMA use (*n*)	19	20	> 0.05
RCA revascularisation (*n*)	14	14	> 0.05

**Fig. 1.**
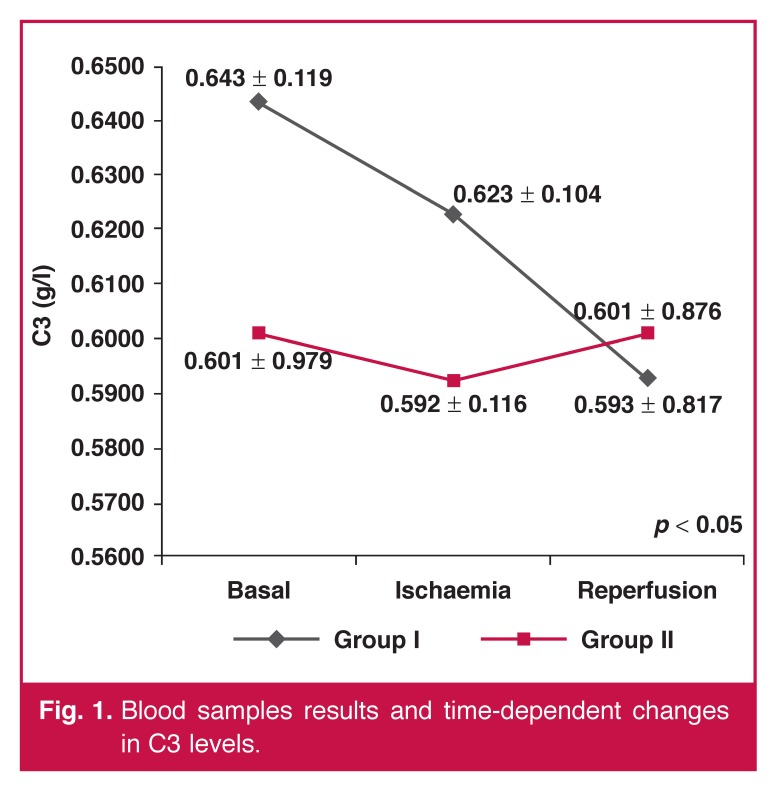
Blood samples results and time-dependent changes in C3 levels.

**Fig. 2.**
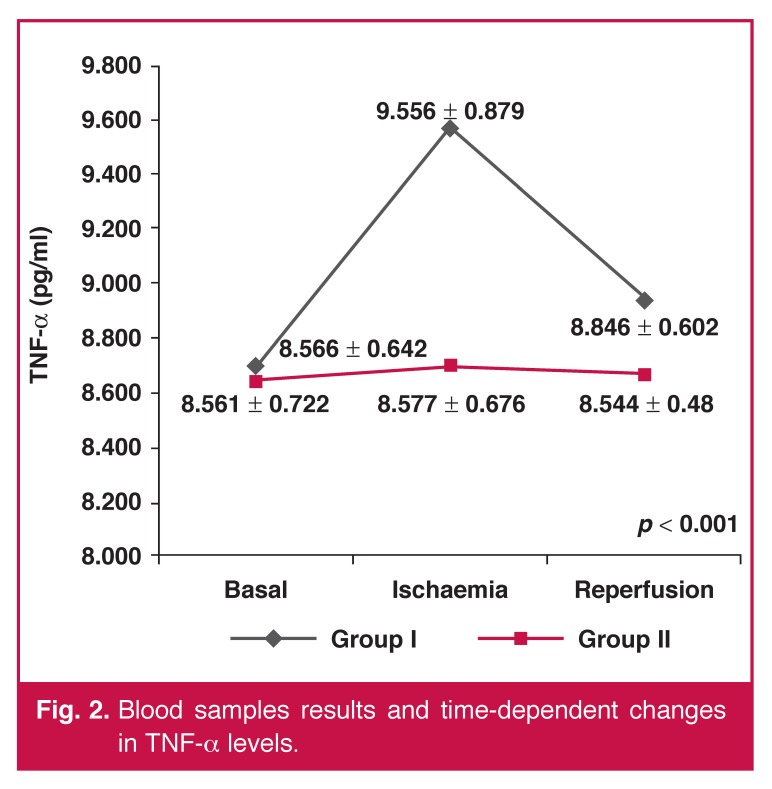
Blood samples results and time-dependent changes in TNF-a levels.

Excluding C3 and TNF-α levels in the time-dependent analysis, there were no statistically significant differences between any blood parameters. When we analysed the changes in C3 and TNF-α levels at every time point and the time-dependent changes, there was a statistically significant difference in favour of group II (C3: *p* < 0.05, TNF-α: *p* < 0.001). The blood sample results and time-dependent changes are given in (able 3 and Figs 1 and 2.

**Table 3:** Blood sample results of the patients.

*Sample type*	*Time*	*Group I (n = 25)*	*Group II (n = 25)*	p*-value*
TnI (ng/ml)	Pre-op	0.314 ± 0.97	0.204 ± 0.35	> 0.05
	Basal	0.326 ± 0.755	0.296 ± 0.251	> 0.05
	Ischaemia	0.602 ± 0.886	0.415 ± 0.337	> 0.05
	Reperfusion	0.938 ± 0.85	0.782 ± 0.372	> 0.05
	8th hour	1.475 ± 0.479	1.09 ± 0.604	> 0.05
	24th hour	1.51 ± 1.343	1.113 ± 0.854	> 0.05
TnT (ng/ml)	Pre-op	0.155 ± 0.497	0.042 ± 0.057	> 0.05
	Basal	0.149 ± 0.420	0.162 ± 0.208	> 0.05
	Ischaemia	0.294 ± 0.603	0.201 ± 0.176	> 0.05
	Reperfusion	0.330 ± 0.65	0.339 ± 0.204	> 0.05
	8th hour	0.495 ± 0.725	0.367 ± 0.235	> 0.05
	24th hour	0.417 ± 0.59	0.331 ± 0.217	> 0.05
Myoglobin (ng/ml)	Pre-op	43.24 ± 48.789	30.368 ± 8.470	> 0.05
	Basal	71.911 ± 40.82	91.028 ± 36.53	> 0.05
	Ischaemia	105.06 ± 53.35	137.02 ± 50.49	> 0.05
	Reperfusion	199.068 ± 88.52	253.22 ± 69.395	> 0.05
	8th hour	156.17 ± 67.164	170.93 ± 81.62	> 0.05
	24th hour	256.63 ± 196.79	233.48 ± 113.867	> 0.05
CK (U/l)	Pre-op	98.28 ± 98.99	91.56 ± 55.49	> 0.05
	8th hour	411.04 ± 130.087	448.6 ± 181.39	> 0.05
	24th hour	624.6 ± 245.75	599.21 ± 256.73	> 0.05
CK-MB (U/l)	Pre-op	13.32 ± 5.49	14.68 ± 4.63	> 0.05
	8th hour	23.84 ± 6.414	24.8 ± 4.76	> 0.05
	24th hour	20.88 ± 8.31	25.2 ± 8.225	> 0.05
LDH (U/l)	Pre-op	381.12 ± 61.42	378.32 ± 91.13	> 0.05
	8th hour	518.12 ± 103.39	538.04 ± 129.84	> 0.05
	24th hour	610.12 ± 110.98	581.84 ± 132.92	> 0.05
C3 (g/l)	Basal	0.6436 ± 0.119	0.601 ± 0.979	< 0.05
	Ischaemia	0.623 ± 0.104	0.592 ± 0.116	< 0.05
	Reperfusion	0.593 ± 0.817	0.601 ± 0.876	< 0.05
C4 (g/l)	Basal	0.115 ± 0.219	0.107 ± 0.06	> 0.05
	Ischaemia	0.114 ± 0.20	0.109 ± 0.52	> 0.05
	Reperfusion	0.109 ± 0.203	0.1068 ± 0.50	> 0.05
TNF-a (pg/ml)	Basal	8.566 ± 0.642	8.516 ± 0.722	< 0.001
	Ischaemia	9.556 ± 0.879	8.577 ± 0.676	< 0.001
	Reperfusion	8.846 ± 0.602	8.544 ± 0.48	< 0.001

There was a statistically significant difference between the groups with regard to spontaneous defibrillation of the heart. Sinus rhythm was spontaneously restored in 20 patients in group II, and in only eight in group I (*p* < 0.01).

Atrial fibrillation postoperatively was seen in six patients in group I, and in only one patient in group II (*p* < 0.05). There was no significant difference between the groups with regard to other rhythm disorders (*p* > 0.05). There were four patients who needed IABP support in group I, whereas no patient needed this in group II (*p* < 0.05).

In group I, seven patients had diaphragm elevation as a consequence of diaphragm paralysis, while none of the patients had diaphragm complications in group II (*p* < 0.01). There was no significant difference between the groups with regard to rates of partial pericardiotomy and LIMA use, which could have caused these diaphragm complications. Comparison of the patients’ data is given in Table 4.

**Table 4:** Comparison of the patients’ data.

	*Group I (n = 25)*	*Group II (n = 25)*	p*-value*
Positive inotropes			
Adrenaline (*n*)	14	11	> 0.05
Dopamine (*n*)	1	0	> 0.05
Dobutamine (*n*)	2	0	> 0.05
ICU			
Intubation time (h)	13.06 ± 3.66	13.28 ± 2.37	> 0.05
ICU stay (h)	29.4 ± 12.28	30.24 ± 20.99	> 0.05
Drainage and transfusions			
Total drainage (ml)	1022 ± 507.12	1004 ± 473.33	> 0.05
Total transfusion (ml)	764 ± 462.7	848 ± 476.20	> 0.05
ECG and echocardiography			
Significant ECG changes (*n*)	0	0	> 0.05
Significant EF changes (*n*)	1	1	> 0.05
Wall motion disorder (*n*)	2	0	> 0.05
Aneurysm formation (*n*)	0	0	> 0.05
Valve dysfunction (*n*)	0	0	> 0.05
Other complications			
Peri-operative MI (*n*)	0	0	> 0.05
Pacemaker need (*n*)	1	0	> 0.05
Low cardiac output (*n*)	1	0	> 0.05
Death (*n*)	1	0	> 0.05
Total hospital stay (day)	8.72 ± 2.42	8.20 ± 1.35	> 0.05

The necessity of using positive inotropes, ICU statistics, drainage and transfusion amounts, ECG and echocardiography changes, hospital length of stay and the other postoperative data are given in Table 5. Overall there was no statistically significant difference between the groups with regard to these categories (*p* > 0.05).

**Table 5:** Other postoperative data.

	*Group I (*n* = 25)*	*Group II (*n* = 25)*	p*-value*
Spontaneous sinus rhythm restoration (*n*)	8	20	< 0.01
Dysrhythmias			
Atrial fibrillation (*n*)	6	1	< 0.05
Ventricular faibrillation (*n*)	1	0	> 0.05
Other (*n*)	1	1	> 0.05
IABP require (*n*)	4	0	< 0.05
Diaphragmatic complications			
Diaphragmatic elevation (*n*)	7	0	< 0.01
Partial pericardiotomy (*n*)	11	13	> 0.05
Pleural effusion (*n*)	3	2	> 0.05

## Discussion

Initially, most cardiac surgeons believed that topical cooling was effective in cardiac performance as it increased myocyte viability and decreased myocardial injury, therefore having a beneficial effect on contractile performance postoperatively. Nikas *et al.*^4^ reported that topical cooling had no effect on myocardial temperature; however, it increased postoperative complications such as diaphragmatic injury, arrhythmias and their consequences by hypothermic myocyte injury. They believed topical hypothermia to be detrimental in cardiac surgical modalities.^4^ After their study, topical cooling become controversial in many cardiac surgery centres.

Minatoya *et al.*^5^ compared cardiac normothermia under normothermic CPB with cardiac hypothermia (with topical cooling) under hypothermic CPB. They reported that hypothermia had more deleterious effects on cardiac myocytes and cardiac contractility. They assessed cardiac enzymes, echocardiography and ECG as an indirect indicator of myocardial damage.^5^ In our study we used pre-operative, intra-operative and postoperative levels of cardiac enzymes and complement factors as an indirect marker of the condition of cardiac myocytes and local myocardial inflammation.

## Blood samples

There were no significant differences between the groups with regard to blood samples, whereas we found a significant difference in time-dependent changes in C3 and TNF-α levels. C3 levels rapidly decreased during the ischaemic and reperfusion periods in group I. In group II, C3 levels decreased minimally in the ischaemic period, and returned to almost baseline values during the reperfusion period (Fig. 1).

Normally, after placing the ACC, ischaemia and the inflammatory period begins, and therefore complement activation starts. With activation of the complement system, C3 is diverted to its subunits, C3a and C3b, and therefore the amount of C3 is rapidly decreased. The decrease is expected to be more severe if inflammation is severe.^6^ In this regard, time-dependent changes in C3 levels showed more inflammatory activity in group I. This may indicate that topical cooling caused more inflammation and therefore more injury to cardiac myocytes.

Similarly, time-dependent changes in TNF-α levels were significant (*p* < 0.001). TNF-α, which is an early reactive cytokine of the inflammatory response, is released during the activation of the inflammatory period. During cardiac surgery, this would occur with ACC.^7^ In our study, TNF-α was rapidly increased with ACC and decreased to almost baseline levels in group I, whereas it showed quite a stable trend in group II (Fig. 2). This result supports the hypothesis of Nikas *et al.*, who showed that local hypothermia caused more inflammation and local injury.^4^

## Defibrillation requirement

Most authors accept that spontaneous restoration of sinus rhythm after aortic declamping is an important indicator of myocardial function.^8^,^9^ Lichenstein *et al.* reported that the rate of spontaneous restoration of sinus rhythm was higher in patients without topical cooling.^9^ In our study, this rate was significantly higher in patients in group II than in those in group I (80 vs 32%, *p* < 0.01).

The need for defibrillation was as follows: in group I, eight patients (32%) needed it once, seven patients (28%) needed it twice, and two (8%) needed it three or more times, whereas in group II, four patients (16%) needed it once and only one patient needed it twice. Therefore, if the requirement for defibrillation were an indicator for myocardial function, group I had poor myocardial function at an early phase of aortic declamping. We believe these results are related to topical cooling because this was the only variable that differed between the groups.

## Incidence of dysrhythmias

Atrial fibrillation (AF) is the most common type of arrhythmia seen after cardiac surgery (32.3%).^10^ The systemic inflammatory response plays an important role in the pathogenesis of these arrhythmias, and several risk factors such as older age, pre-operative history of AF, chronic obstructive pulmonary disease (COPD), long intubation time, long ACC time, renal insufficiency, and high amounts of drainage and transfusion were found to be responsible for postoperative AF.^10^-^14^

In our study, there was a statistically significant difference between the two groups for AF rates (six patients in group I vs one patient in group II, *p* < 0.05). The patients were randomised homogenously into the groups according to these risk factors. Therefore, based on these results, we consider that there was a direct relationship between topical cooling and postoperative AF.

## IABP requirement

In the literature, there are not many studies on the association between IABP and topical myocardial cooling. In one study, which compared the effects of cardiac hypothermic and normothermic techniques, Calafiore *et al.* reported that there was no statistically significant difference between the type of technique used and the necessity for IABP.^15^

In our study, IABP was required in four patients (16%) in group I, while none of the patients required it in group II (*p* < 0.05). Although these results were statistically significant, we are not certain of the relationship between these two factors because indications for IABP are standardised worldwide in daily practice. However, it is usually dependent on the surgeon’s decision. We believe there was no definite association between necessity for IABP treatment and topical myocardial cooling.

## Diaphragm pathology

There was a statistically significant difference in diaphragm paralysis between the groups in our study (*n* = 7, 28% vs *n* = 0, 0%, *p* < 0.01). We believe the main pathogenesis was phrenic nerve injury. The phrenic nerve originates from the anterior horn of C3–C6, runs down the posteromedial part of the internal mammarian artery at the entrance of the thoracic cavity, spreads to the lateral surface of the pericardium and then reaches the diaphragm muscles.

The main artery of this nerve, which may be damaged during LIMA harvesting due to its proximity to the internal mammarian artery, is the pericardiophrenic artery.^16^ This nerve can be damaged during LIMA harvesting and/or partial pericardiotomy, which is performed for tunnelling to the LIMA pedicle. Furthermore the damage may occur because of cold application. This type of injury primarily depends on nerve demyelisation and is usually reversible in one year.^4^ Phrenic nerve injury after cardiac surgery is reported at 2–17%.^17^

In another study, Nikas *et al.* compared diaphragm paralysis in 505 patients undergoing cardiac surgery with and without topical hypothermia, and they found similar result to those in our study. In this study, 25% of patients with topical hypothermia and 2% of those without topical hypothermia had diaphragm paralysis (*p* < 0.0001).^4^

In our study, the first intercostal artery was devascularised with a haemostatic clip and without cauterisation for maximal care of the phrenic nerve during LIMA harvesting. Partial pericardiotomy was performed carefully on 11 patients in group I and 13 patients in group II (*p* > 0.05). These patients were compared in each group for an association between possible phrenic nerve injury and partial pericardiotomy. There were no correlations between pericardiotomy, LIMA harvesting and phrenic nerve injury. Therefore cold injury seemed to be responsible for phrenic nerve injury and thus diaphragm paralysis.

## Other parameters

There was no statistically significant difference for necessity of positive inotrope, and drainage and transfusion amounts between the groups. Abacilar *et al.* reported a positive correlation between TNF-α level and postoperative mediastinal drainage amount.^2^ We found a similar relationship between these two parameters in our study, although not statistically significant.

Robicsek *et al.* reported that cardiac myocyte dehydration due to relative hyperosmolarity secondary to hypothermal iced saline caused myocardial functional disorder, and with the progression of this process, cell death may occur in time.^18^ In another study, Conno *et al.* reported that topical cooling with iced saline may cause epicardial oedema via a hypothermic–hypo-osmolar effect and may create temporary ST-segment anomalies on ECG without myocardial functional disorders.^19^ We did not see any significant abnormalities in ECG or echocardiography in our study.

Hamulu *et al.* reported that pre-operative cardiac dysfunction was an important risk factor for heart surgery and reported that mortality and morbidity rates were almost higher in patients with pre-operative EF < 35%.^20^ We excluded the patients with < 35% EF from our study for realistic results.

None of our patients had major complications such as cerebrovascular events, renal insufficiency or infection. A 58-year-old male patient in group I, treated for diabetes mellitus and hypertension, was dead on postoperative day 18 due to cardiac and respiratory arrest at home. When we compared the risk factors of peri-operative mortality such as age, gender, concomitant diseases, myocardial function, EF and surgical parameters,^21^ we did not find any correlation between this patient and the remaining patients in either group.

## Conclusion

Although this was a randomised, controlled, prospective study, due to the small number of patients, it was not possible to make generalised comments regarding the results. Therefore, there is a need to perform more randomised studies with larger patient numbers.

Since topical hypothermia was the only variable in the present study, it was shown to increase inflammatory activity. Therefore topical hypothermia had deleterious effects on the postoperative course, which affects postoperative morbidity and patient outcome.
